# The British Journals: Anatomy and Physiology

**Published:** 1841-07

**Authors:** 


### III. THE BRITISH JOURNALS.

(FOR THE QUARTER ENDING MAY 30, 1841.)

### ANATOMY AND PHYSIOLOGY.

*On the Relation between Muscular Contractility and the Nervous System.* By JOHN REID, M.D.

THIS short paper contains some very interesting experiments upon this *quæstio vexata* of physiology, devised and executed for the purpose of removing some of the grounds of the objections recently made by Müller, Dr. M. Hall, and others, against the Hallerian doctrine of the independent contractility of muscular fibre. Dr. Reid had long ago ascertained, that the contractility of muscles whose nerves had been divided, would return in frogs after having been exhausted; and this not once only, but several times. He has now established the same fact by experiments on rabbits; and has thus removed the objection made to his former results, on the score of their being afforded by cold-blooded animals. Hence it would appear that the contractility cannot be derived from the nervous system, but must be a *vis insita* of the muscle. It has been urged by Müller, in support of the neurological doctrine, that muscles whose nerves have been for some time divided lose their contractility; and it has been replied that the disordered state of their nutrition, consequent upon their want of use (which is shown by microscopical examination to change the structure of muscular fibre), was sufficient to account for facts of this kind. Dr. Reid has very ingeniously proved this explanation to be the true one. In a rabbit, whose sciatic nerve had been divided on one side, the contractility of the muscles of the leg was nearly lost at the end of seven weeks; these muscles were found to weigh only 170 grains, whilst those of the sound limb weighed 327 grains, or nearly double; and the tibia and fibula had diminished from 89 grains to 81. A still more decisive experiment was next performed. The spinal nerves were cut across in the lower part of the spinal canal of four frogs, and both posterior extremities were thus insulated from the nervous centres. The muscles of one of the paralysed limbs were daily exercised by a weak galvanic battery, while those of the other limb were allowed to remain quiescent. This was continued for two months; and at the end of that time, the muscles of the exercised limb retained their original size and firmness, and contracted vigorously; while those of the quiescent limb had shrunk to one-half their former bulk, not yet losing, however, their irritability.

Dr. M. Hall has expressed the opinion that the spinal cord is the seat of irritability; grounding this view upon the fact, that, if one limb be paralysed to the influence of volition by dividing the spinal cord, but retain its connexion with as much of the latter as suffices for reflex action, whilst the other is cut off from all communication with the nervous centres,—the muscles of the former will retain their irritability, when those of the latter have lost theirs. Dr. Reid justly remarks upon this, that the muscles of the former limb would be frequently in action by the excitement of contact, &c. during the ordinary movements of the animal; and that thus their nutrition would be maintained, and their irritability preserved, as when differently excited to action in the last experiment; and, further, that the stimulus of a galvanic current in water, the test employed by Dr. Hall, would act upon the former, not only by directly exciting the muscles, but by reflected operation through the spinal nerve, so that it would occasion more powerful contractions in that limb than in the other, on the muscles of which it had only one mode of action. Moreover, the doctrines of Dr. Hall and Müller are totally irreconcileable with Dr. Reid's experiments; so that the original statements of Haller must still be preferred to them.

*Edinburgh Monthly Journal of Medical Science. May,* 1841.

---

*On the Order of Succession in which the Vital Actions are arrested in Asphyxia.* By John Reid, M.D., F.R.C.P.E., &c.

Two questions, which had not been satisfactorily replied to by preceding experimenters, are proposed for investigation in this valuable paper; and they are treated with the skill constantly manifested by its author in the elucidation of difficult subjects of physiological enquiry.

The experiments of Drs. Williams and Kay demonstrated, that in Asphyxia the circulation is first brought to a stand by some impediment to its free passage through the lungs; but they did not afford ground for determining whether this impediment results from the cessation of the usual respiratory movements of the chest, which are known to have an important influence on the movement of the blood, or whether it is to be attributed to the arrestment of the usual chemical changes between the blood and atmospheric air. The latter view, upheld by Dr. Alison on general grounds, was also supported by the fact ascertained by him, that, if animals be asphyxiated by breathing an atmosphere of nitrogen, an accumulation of blood on the right side of the heart commences before the respiratory movements have been seriously weakened. Much more conclusive evidence has been afforded, however, by Dr. Reid's experiments. He fixed a tube with a stop-cock in the trachea, and applied a hemadynamometer to the femoral artery. By shutting the stop-cock, the respiratory process was suspended; and upon inspecting the hemadynamometer, the very unexpected result became apparent, that, when the asphyxia was proceeding to the stage of insensibility, and the attempts at respiration were become few and laboured, and the blood in an exposed artery was quite venous in its character, the pressure upon the walls of the artery was much *greater* than when the animal was breathing freely. Upon applying a similar test to a vein, however, it was found that the pressure was proportionally diminished; whence it became apparent that there was an unusual obstruction to the passage of the venous blood through the systemic capillaries, a fact of much importance in the general theory of the circulation, and also explaining how it is that some amount of blood is generally retained on the left side of the heart in asphyxia. After this period, however, the mercury began to fall steadily, and at last rapidly, in consequence of the diminished force of the heart; and its downward progress was not at all arrested, by causing the animal to breathe nitrogen, a bladder of which gas was screwed to the stop-cock. But if atmospheric air was then admitted, the mercury rose very speedily; showing that it was by the chemical influence of the oxygen that the force of the arterial circulation was restored, and not by the respiratory movements, which had been previously renewed in the nitrogen, without any accelerating influence. These experiments, then, fully confirm the view that the stagnation of blood in the capillaries of the lungs is not due to a mechanical cause, but to the interruption of the chemical processes essential to its movement through the capillaries, by the exclusion of the necessary element. And they add the important fact, confirmatory of the same general doctrine, —that venous blood will not readily pass through the systemic capillaries, the changes to which it should there minister being prevented by the depravation of its own character.

The second question investigated by Dr. Reid relates to the cause of the arrestment of the sensorial functions in Asphyxia. It is well known that Bichat considered this to be due to the circulation of venous blood through the brain; but that Dr. Kay, on the other hand, attempted to prove that it resulted from an absolute deficiency of the circulating fluid. Dr. Reid shows the unsatisfactory nature of the experiments of each of these enquirers; and proves satisfactorily, to our minds at least, that the sensorial functions are greatly impaired, before the supply of blood has sustained any considerable diminution,—in fact, whilst its pressure on the coats of the arteries is greater than ordinary, from the cause just explained; and as, at this time, its character has been almost reversed, we can have little hesitation in assenting to Bichat's opinion of the cause of the phenomenon. "We do not however," says Dr. Reid, "maintain that venous blood exerts any noxious influence upon the functions of the nervous texture; but believe that its effects are solely to be attributed to the want of the proper excitation of this organ; for, when the circulation of arterial blood is renewed, its functions rapidly manifest themselves, provided that this be done within a given time."

"We believe, then, that, in Asphyxia, the order of succession in which the vital processes are arrested is as follows:—The [partly] venous blood is at first transmitted freely through the lungs, and reaches the left side of the heart, by which it is driven through all the textures of the body. As the blood becomes more venous, its circulation through the vessels of the brain deranges the sensorial functions, and rapidly suspends them, so that the individual becomes unconscious of all external impressions. The functions of the medulla oblongata are enfeebled about the same period that the sensorial functions are arrested, but are not fairly suspended for some time after. Immediately after the sensorial functions are suspended, and the blood has become still more venous, it is transmitted with difficulty through the capillaries of the lungs, and consequently begins to collect in the right side of the heart. A smaller quantity of blood must now necessarily reach the left side of the heart; and thus diminution of the quantity of blood sent along the arteries, conjoined with its venous character, and the ultimate arrestment of the circulation, being circumstances incompatible with the manifestation of vitality in the other tissues of the body, in general death is sooner or later induced."

*Edinburgh Medical and Surgical Journal. April,* 1841.

